# Pharmacophore-Based Virtual Screening Toward the Discovery of Novel Anti-echinococcal Compounds

**DOI:** 10.3389/fcimb.2020.00118

**Published:** 2020-03-20

**Authors:** Congshan Liu, Jianhai Yin, Jiaqing Yao, Zhijian Xu, Yi Tao, Haobing Zhang

**Affiliations:** ^1^Key Laboratory of Parasite and Vector Biology, Chinese Center for Disease Control and Prevention, National Center for International Research on Tropical Diseases, WHO Collaborating Centre for Tropical Diseases, National Institute of Parasitic Diseases, MOH, Shanghai, China; ^2^Drug Discovery and Design Center, Shanghai Institute of Materia Medica, Chinese Academy of Sciences, Shanghai, China

**Keywords:** echinococcosis, *Echinococcus multilocularis*, pharmacophore modeling, virtual screenings, *in vitro* drug screen, cytotoxicity, *E. multiloculari*s-infected mice, pharmacokinetics analysis

## Abstract

Echinococcosis is a serious helminthic zoonosis with a great impact on human health and livestock husbandry. However, the clinically used drugs (benzimidazoles) have a low cure rate, so alternative drugs are urgently needed. Currently, drug screenings for echinococcosis are mainly phenotype-based, and the efficiency of identifying active compounds is very low. With a pharmacophore model generated from the structures of active amino alcohols, we performed a virtual screening to discover novel compounds with anti-echinococcal activity. Sixty-two compounds from the virtual screening were tested on *Echinococcus multilocularis* protoscoleces, and 10 of these compounds were found to be active. After further evaluation of their cytotoxicity, S6 was selected along with two active amino alcohols for *in vivo* pharmacodynamic and pharmacokinetic studies. At the two tested doses (50 and 25 mg/kg), S6 inhibited the growth of *E. multilocularis* in mice (14.43 and 9.53%), but no significant difference between the treatment groups and control group was observed. Treatment with BTB4 and HT3 was shown to be ineffective. During the 28 days of treatment, the death of mice in the mebendazole, HT3, and BTB4 groups indicated their toxicity. The plasma concentration of S6 administered by both methods was very low, with the C_max_ being only 1 ng/ml after oral administration and below the detection limit after intramuscular administration. In addition, the plasma concentrations of BTB4 and HT3 *in vitro* did not reach high enough levels to kill the parasites. The toxicities of these two amino alcohols indicated that they are not suitable for further development as anti-echinococcal drugs. However, further attempts should be made to increase the bioavailability of S6 and modify its structure. In this study, we demonstrate that pharmacophore-based virtual screenings with high drug identification efficiency could be used to find novel drugs for treating echinococcosis.

## Introduction

*Echinococcus granulosus* and *E. multilocularis* are the most important *Echinococcus* species currently affecting humans; in the larval stages, these species cause cystic echinococcosis (CE) and alveolar echinococcosis (AE), respectively (Eckert et al., 2001). If the eggs in the feces of definitive host (dogs or foxes) are ingested by humans, the metacestode cysts can asexually proliferate mainly in the liver and lungs (Moro and Schantz, 2009). CE occurs worldwide, while AE is confined to the Northern Hemisphere (WHO/Department of Control of Neglected Tropical Diseases, 2013). The growth of these parasites in patients is slow, and until the parasites grow to an extent that triggers clinical signs, which takes many years, their growth remains asymptomatic (Moro and Schantz, 2009). The drugs currently available in clinical settings are primarily limited to benzimidazoles (BMZ), albendazole and mebendazole, and the chemotherapy usually lasts 3 to 6 months but can last even longer (Lacey, 1990; Hemphill and Muller, 2009). Unfortunately, the efficacy of these drugs is only ~30%, with more than 40% of patients showing side effects (such as headache and abnormal liver function; Davis et al., 1986, 1989; Eckert et al., 1995). No other medications to treat echinococcosis have been approved in the last 30 years; hence, it is essential to find alternative chemotherapy strategies for treating this disease.

The development of drugs for echinococcosis, as a neglected disease, is of very limited interest to the pharmaceutical industry. Hence, none of the candidate alternative drugs or compounds tested against *in vitro* or *in vivo* models of *Echinococcus* spp. were first designed for the treatment of CE and AE (Siles-Lucas et al., 2018). For decades, most studies have focused on BMZ and new formulations to improve solubility or on the repurposing of clinical drugs (antitumour, antiviral, and antibiotic drugs; Siles-Lucas et al., 2018). Among the developed compounds, a limited number of drugs (isoprinosine Sarciron et al., 1992, mefloquine Rufener et al., 2018, tamoxifen Nicolao et al., 2014, etc.) have been shown to be effective in infected animal models. All the drug studies mentioned above are based on phenotypic screenings, which depend on the experience of the researchers. Hence, this screening method is essentially subjective and time consuming, resulting in low screening efficiencies. Recently, algorithms and imaging systems have been developed to measure the effects of drugs on the activities of *E. multilocularis* protoscoleces (PSCs). This advance has greatly increased screening efficiencies and reduced the requirement for experienced researchers; thus, it is considered an ideal screening method (Ritler et al., 2017). However, this improved test method has not substantially change the anti-echinococcal drug development pathway. The selection of candidate drugs for screening is limited and blind, and the structures of effective drugs and compounds are not fully exploited, resulting in inefficient use of research time and data.

Computational techniques have become the most effective methods in drug discovery and development. Among such methods, homology modeling, molecular docking, pharmacophore modeling and structure-based virtual screenings have been successfully applied in drug discovery. The virtual screening of a large number of compounds can identify the best molecular structure for combination with a specific biological target. In addition, analysis of structure-activity relationships (SARs) is the basis of designing new compounds, making structural modifications and predicting unknown active compounds (Shah et al., 2015; Ganesan and Barakat, 2017). For example, to find novel and potent anticancer agents, structure-based pharmacophore models based on promising cancer therapy drug targets (such as matrix metalloproteinase-2, histone deacetylase-6, protein kinase B-beta, and glycogen synthase kinase-3) have been developed and validated. Many compounds identified in virtual screenings have shown potential efficacies, and various chemical scaffolds have been proposed as potential leads for the further development of new anticancer agents (Crisan et al., 2017; Akhtar et al., 2018). Moreover, computer-aided drug design has been applied to the discovery of anti-parasitic drugs. Using pharmacophore-based virtual screening and molecular dynamics and molecular docking approaches, promising drug targets and hits are identified (Pavadai et al., 2016; Vyas et al., 2016; Kagami et al., 2017; Araujo et al., 2018). Among the predicted active compounds, some were shown to have anti-malarial activities *in vitro* and *in vivo* (Pavadai et al., 2016; Vyas et al., 2016). Leucyl-tRNA synthetase and pteridine reductase of trypanosomatid parasites were used as potential targets for screening novel anti-trypanosomatid compounds, and new potential inhibitory scaffolds were predicted; however, further validation is needed (Dube et al., 2012; Zhao et al., 2012). The development of a pharmacophore model for ligands of *S. mansoni* purine nucleoside phosphorylase (SmPNP) as well as a pharmacophore-based virtual screening approach led to the identification of three thioxothiazolidinones with substantial *in vitro* inhibitory activity against SmPNP (Postigo et al., 2010). However, there have been no reports of applying these methods in drug discovery for the treatment of echinococcosis.

In our previous study, we reported a series of amino alcohols with potential anti-echinococcal effects (Liu et al., 2018). The aim of this study was to screen potential compounds by pharmacophore models based on the structure of these active amino alcohols. The ligand- and structure-based pharmacophore models were developed to identify compounds with potential anti-echinococcal activity. HipHop-Hypo03 was selected as the most promising hypothesis, as it includes one positive ionizable (PI) moiety, one hydrophobic aromatic (HAR) moiety, one hydrogen bond donor (HC) and two hydrophobic aliphatic (HAL) moieties. Subsequently, these pharmacophore hypotheses were used in the virtual screening of ZINC databases to identify potential active compounds. The screened compounds were then tested on *E. multilocularis* PSCs *in vitro*. The *in vivo* efficacy and pharmacokinetics of one of the screened compounds together with two amino alcohols were determined.

## Materials and Methods

### Pharmacophore Model

#### Data Collection and Preparation

Based on our previous research results (Liu et al., 2018), six structurally diverse amino alcohols active against *E. granulosus* PSCs and germinal cells at 20 μg/ml were used as the training set to generate common feature-based pharmacophore models. The molecular properties of these compounds are listed in Table 1. Thirty-four amino alcohols were selected as the test set, among which 17 had anti-echinococcal effects (labeled with A+ID numbers) and 17 were inactive (labeled with ID numbers). All the molecules were prepared and optimized using Discovery Studio (DS) v4.1.

**Table 1:** Chemical structures and molecular properties of the compounds in the training set.

**Structure**	AlogP^*^	**Molecular weight**	Num-H^**^ acceptors	**Num-H donors**	**Num-H rotatable bonds**	**Molecular polar surface area**
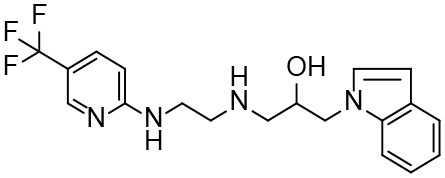	3.209	378.391	9	4	3	62.11
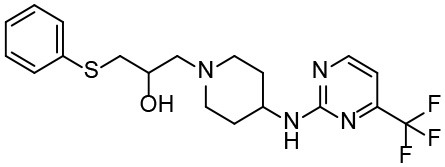	3.069	412.472	8	6	2	86.58
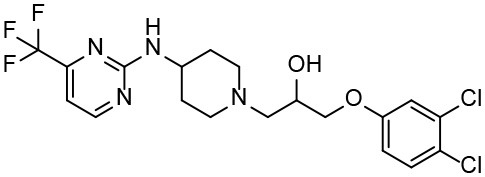	3.84	465.297	8	6	2	70.51
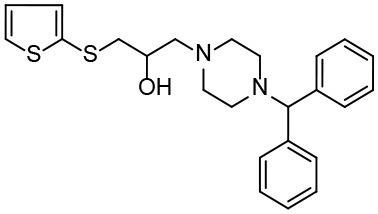	4.939	424.622	8	4	1	80.25
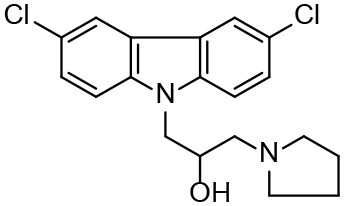	4.947	363.281	4	2	1	28.4
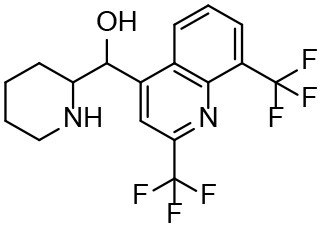	4.302	378.312	4	3	2	45.15

* Alop, partition coefficient;

** *Hum-H, number of H-bond*.

#### Pharmacophore Model Generation

HipHop pharmacophore models were generated from a set of six active amino alcohols with promising anti-echinococcal activities (Table 1). The principal value 2 was set for all the ligands and the maximum-omit feature was set to 0. Before building the HipHop pharmacophore model, the pharmacophore module “Feature Mapping” was used to identify the important chemical features of the training set compounds. Then, hydrogen bond acceptor (HBA), hydrogen bond donor (HBD), lipid hydrogen bond acceptor (LHBA), hydrophobic feature (HC), hydrophobic aliphatic (HAL), hydrophobic aromatic (HAR), positive ionizable (PI) and aromatic ring (AR) were selected as the building blocks for generating the pharmacophore model. To obtain diverse conformations, 255 conformations within a 20 kcal/mol energy threshold were generated using the “BEST” function. The final pharmacophore models with key common chemical features were ranked by fit value and are listed in Table 2.

**Table 2:** Chemical features of the ten hypotheses generated from the six active amino alcohols.

**Hypothesis**	**Features**	**Rank**
01	PXZZD	75.332
02	RPZZH	73.804
03	PXZZD	72.884
04	RPZZA	72.604
05	PXZZD	72.571
06	RPZZD	71.987
07	RZZHH	70.926
08	PXZZH	70.909
09	PXZZH	70.909
10	PXZZD	70.849

*P (positive ionizable), X (hydrophobic Aromatic), Z (hydrophobic aliphatic), D (hydrogen bond donor), H (hydrophobic feature), A (hydrogen bond acceptor), R (ring aromoatic)*.

#### Validating the Pharmacophore Model

The hypothesis of about pharmacophore is validated based on the fit results of obtained with test sets. The test sets (Supplementary Figure 1), including active compounds and inactive compounds, were prepared using the same protocol as that for the training set. Then, the “Ligand Profiler module” was used to determine whether the hypothesis was able to identifiy the active compounds according to the Fit Value.

### Pharmacophore-Based Virtual Screening

The validated pharmacophore model HipHop-Hypo03 was used to screen compounds. Approximately 40,000 small molecules were obtained from the ZINC database (www.zinc.docking.org) and subjected to virtual screening. All these compounds were optimized in DS2.5, and HipHop-Hypo03 was used to screen compounds by using the **Screen Library module** of DS2.5. The number of conformations was set to **255**, while the conformation method was set to **BEST**. The remaining parameters were set to the default values. Lipinski's Rule of Five and Veber's drug-likeness rule (Lipinski et al., 2001; Veber et al., 2002) were applied for the subsequent phase of screening. Then, the compounds with good Fit Value were purchased and validated by determining their *in vivo* and *in vitro* effects on *E. multilocularis*.

### Chemicals and Reagents

The screening compounds were purchased from Enamine Ltd. (Kievska region, Ukraine) and had >90% purity. All culture media and reagents were purchased from Gibco-BRL (Zurich, Switzerland); mebendazole and other reagents were from Sigma (St. Louis, MO, USA). The CCK-8 kit was purchased from Dojindo (Tokyo, Japan). Stock solutions of all the compounds for *in vitro* drug screenings were prepared in DMSO at 20 mM, and on the day of treatment, the working solutions were prepared by freshly diluting the stock solution with DMSO to the appropriate concentration. For oral administration and pharmacokinetic analysis, all the compounds were suspended in 1% tragacanth solution.

### Parasites and Animals

*E. multilocularis*-infected animals [BALB/c mice (SLAC Laboratory Animal Center)] were maintained by serial i.p. transplantation passages through for 6 months, the details can be found in our published paper (Liu et al., 2019). Eighty infected BALB/c mice were prepared for *in vivo* treatment experiments. Parasite materials were isolated from the peritoneal cavity of the infected animals and homogenized with Hanks' balanced salt solution (HBSS). Then, after passing the parasite homogenate through a 60-mesh sieve, the *E. multilocularis* PSCs were collected and rinsed 5~8 times. The viability of the PSCs was determined by the methylene blue exclusion method (Liu et al., 2018), and PSCs with > 95% viability were used in the following experiments. The PSCs were cultured in RPMI 1,640 medium supplemented with 10% CS and antibiotics (100 U/ml penicillin G and 100 μg/ml streptomycin) at 37°C in 5% CO_2_.

### Ethics Statement

Animal care and all animal procedures were carried out in compliance with the Guidelines for the Care and Use of Laboratory Animals produced by the Shanghai Veterinary Research Institute. The study was approved by the Ethics Committee of the National Institute of Parasitic Diseases, Chinese Center for Disease Control and Prevention. The license number was IPD-2014-2.

### *In vitro* Drug Screen With *E. multilocularis* Protoscoleces

*In vitro* treatment of PSCs was performed in 96-well microtiter plates (Costar, USA) containing 100 PSCs per 200 μl. A solution of the test compound (6.25~100 μM) was added to each well. Nitazoxanide and mebendazole (25 μM) were used as positive controls, while RPMI 1640 medium and RPMI 1640 with 0.25% DMSO were used as negative controls. The plates were incubated under the culture conditions for 72 h, and then the viability of the PSC was tested by the methylene blue exclusion method (Liu et al., 2018). All experiments were carried out in triplicate and repeated at least twice. The LC_50_ values were calculated by the probability unit method with SPSS version 17.0.

### Cytotoxicity Test

Cytotoxicity was determined using Chang liver cells and A172 cells in DMEM with 10% FBS as previously described (Liu et al., 2018). The final concentrations of the test compounds were 3.125~50 μM for A172 cells and 6.25~100 μM for Chang liver cells. After 72 h, the cell viabilities were determined using a CCK-8 kit. The concentration that induced 50% cell toxicity (Tox_50_) was then calculated.

### Compound Efficacy in *E. multilocularis-*Infected Mice

Eighty infected BALB/c mice were randomly divided into eight groups (*n* = 10 per group) and administered one screened compound (S6) and two amino alcohols (BTB4 and HT3) at 25 and 50 mg/kg/day (S6/BTB4/HT3-25 and S6/BTB4/HT3-50 by oral gavage, the dosage corresponded to that of mebendazole) and mebendazole (MBZ, the most effective drug for the treatment of infected animals) at 25 mg/kg/day (MBZ-25) for 28 consecutive days. The remaining mice were orally administered 1% tragacanth solution (1% TRA) as a control. All of the mice were euthanized 2 weeks after finishing the treatment, and all of the cysts were isolated and weighed. The efficacy of the treatments was assessed based on the mean cyst weight as previously described (Liu et al., 2015).

### Pharmacokinetics Analysis

To study the relationship between pharmacokinetic characteristics and treatment efficacy *in vivo*, compounds were administered by oral (50 mg/kg) and intramuscular (12.5 mg/kg) methods to healthy BALB/c mice (22~22 g). Subgroups of 3 mice each were bled at 0.25, 0.5, 1, 2, 4, 8, 16, 24, 32, and 48 h post-administration. Then, 300 μl samples of plasma were collected and centrifuged at 2,500 g for 15 min. The compounds in the plasma were extracted on Oasis HLB cartridges (Waters, Massachusetts, USA) with praziquantel as the internal standard. The HPLC-HRMS system used for separation and analysis consisted of an Accela 1250 pump, a PAL HTC autosampler, an Accela PDA detector and an Xcalibur data system (Thermo Fisher Scientific, Waltham, MA, USA). Compounds were separated on a 3-μm Waters Atlantis 2.1 mm × 100 mm column (Massachusetts, USA) with a mobile phase containing methanol and formic acid at a flow rate of 0.35 ml/min. The data were analyzed by the non-compartmental model present in DAS 2.0 (Drug Analysis System, Shanghai University of T.C.M, China). Detailed pharmacokinetic experimental methods, including a representative HPLC-MS chromatogram, a standard curve, and a plot used to confirm precision, are shown in the Supplementary Data.

### Data Analysis

The LC_50_ and Tox_50_ values were calculated by the probability unit method with SPSS version 17.0. The differences in mean cyst weight were analyzed by ANOVA in SPSS 17.0, and *P* < 0.05 was considered statistically significant.

## Results

### Pharmacophore Model Generation, Validation, and Virtual Screening

**Figure 1:**
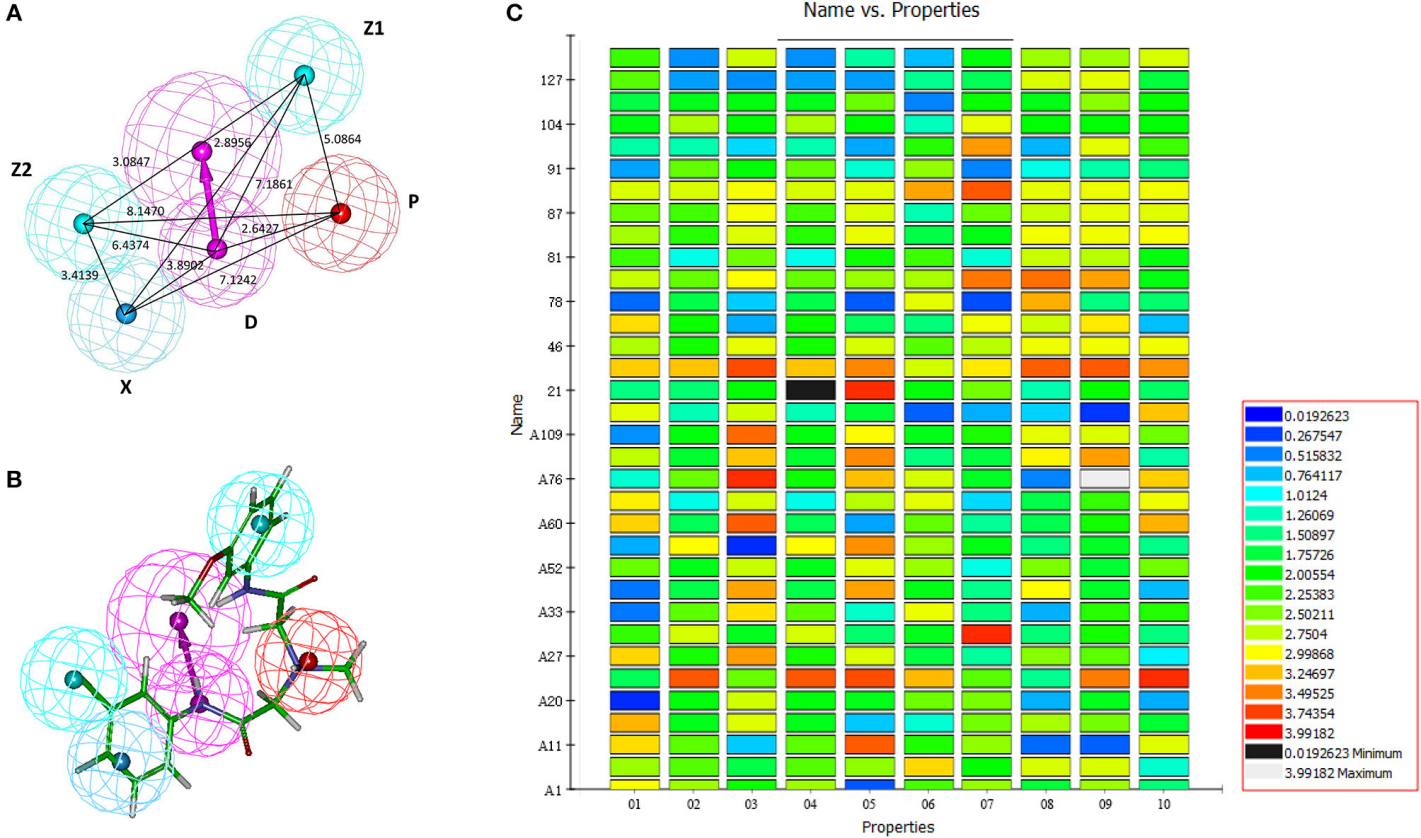
Generation of the HipHop pharmacophore. **(A)** The HipHop-Hypo03 chemical features. The color of the pharmacophore features, namely, PI, HAR, HAL and HBD, are red, blue, cyan, and magenta, respectively. **(B)** S6 fit to HipHop-Hypo03. **(C)** The heat map of the 10 hypotheses in the test.

After the 10 HipHop models were generated, a test set composed of active and inactive amino alcohols was used to select the best model (Figure 1C). The pharmacophore model HipHop-Hypo03 was considered the best chemical hypothesis because the model was better at distinguishing active from inactive compounds. As shown in Figure 1B, the Fit Value of the 11 active compounds were above 3.0, and higher than the Fit Value of most of the inactive molecules, which were distributed in the blue and green areas. As shown in Figure 1B, this pharmacophore included one PI moiety, one HAR moiety, two HAL moieties and one HBD moiety (Figure 1A).

After virtual screening based on this pharmacophore, the fitness scores of 176 compounds were >4.0. Among these compounds, 62 (Supplementary Table 1) were purchased, and their activities against parasites cultured *in vitro* were evaluated.

### Evaluating the Activities of Candidate Compounds on *E. multilocularis* Protoscoleces *in vitro* and Their Cytotoxicities

**Figure 2:**
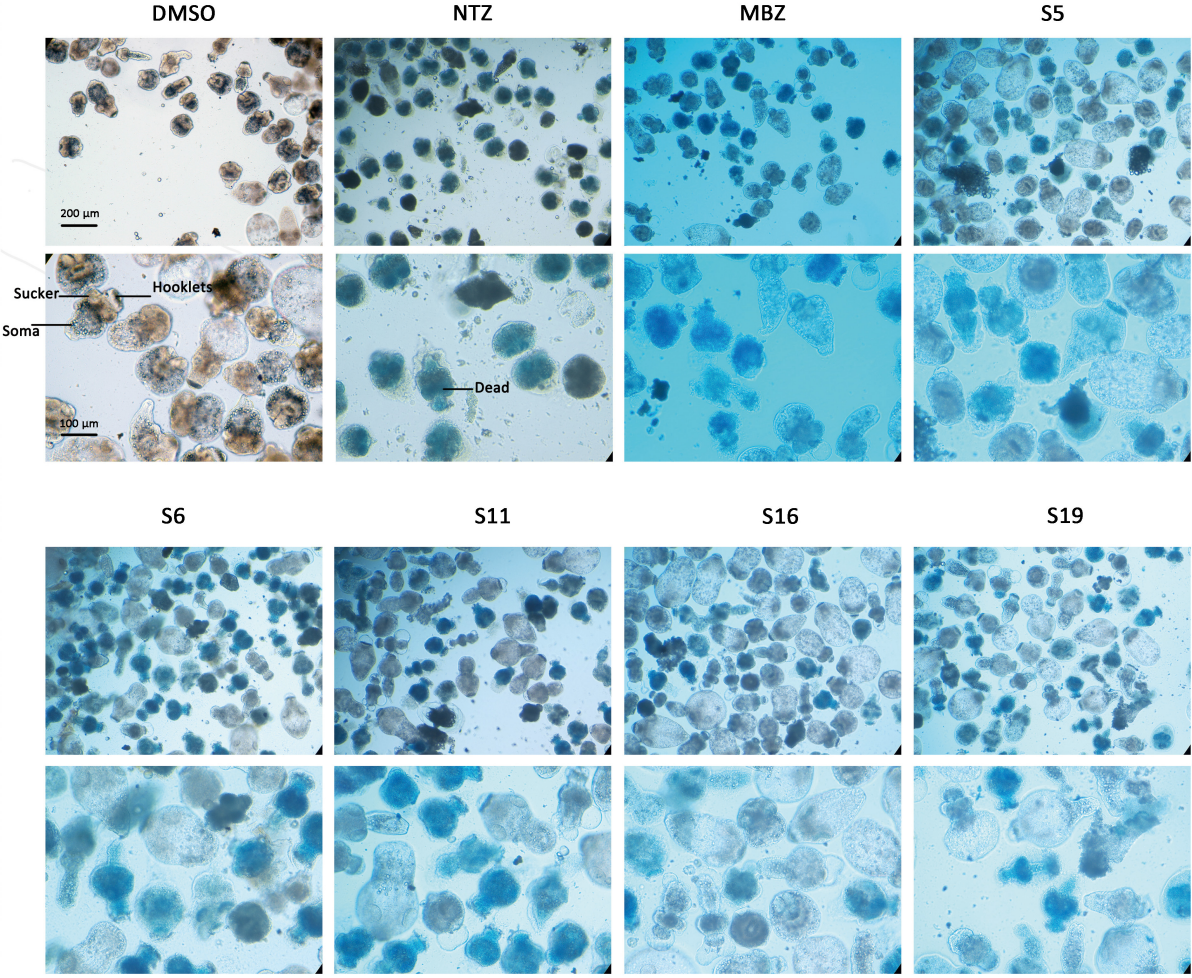
Morphology changes in *Echinococcus multilocularis* protoscoleces after incubation with the active compounds at 25 μM for 3 days. The normal protoscoleces in DMSO had intact soma, sucker and orderly arranged hooks. The drug treated parasites showed partial collapse and disruption of the inner structure, the detachment of hooks and staining with methylene blue.

The effects of the 62 compounds on *E. multilocularis* PSCs were evaluated, and at 100 μM, 10 of the 62 compounds resulted in 100% parasite death in 3 days. Next, solutions of these compounds at a series of concentrations were tested on *E. multilocularis* PSCs to determine their LC_50_ values. The LC_50_ values of the active compounds were 16.99~20.45 μM. As shown in Figure 2, the normal PSCs in DMSO had intact soma, and suckers and orderly arranged hooklets. Exposure to the compounds resulted in morphological damage to the parasites, including irregularities in their internal structure and the detachment of the hooklets from the rostellum. The *E. multilocularis* PSCs stained by methylene blue were dead (Figure 2).

The cytotoxicity of these compounds was also determined using a CCK-8 kit. The low cytotoxicities of S6, S11, S16, and S19 indicated good druggability. In addition, these active compounds (Table 3) were subjected to a cytotoxicity assay using the CCK-8 kit and A172 cells, because of the similarity of this parasite with cancer cells. The results showed that compounds S5, S6, S11, S16, S19, and S28 inhibited the proliferation of cancer cells (A172 cells).

**Table 3:** *In vitro* effects of amino alcohols against *E. multilocularis* protoscoleces, cancer cells (A172 cells) and noncarcinogenic mammalian cells (Chang liver cells).

**Compounds**	**Structure**	**LC_**50**_ (μM)**	**Tox**_****50****_ **(μM)**	
		***E. multilocularis* PSC**	**A172 cell**	**Changliver cell**
S1	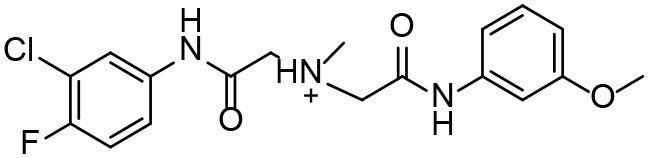	20.45 ± 0.51	ND	43.98 ± 1.38
S5	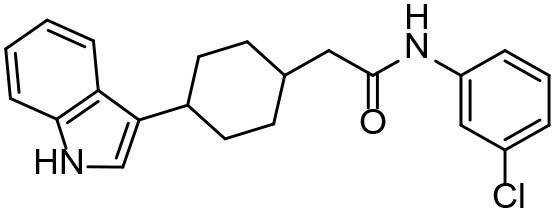	18.60 ± 1.00	11.98 ± 0.91	42.44 ± 0.34
S6	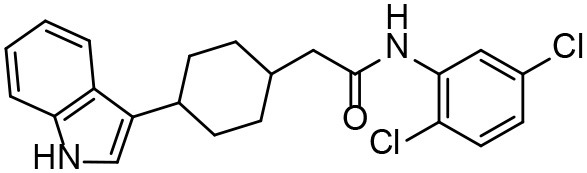	16.99 ± 0.93	11.00 ± 5.00	^*^
S11	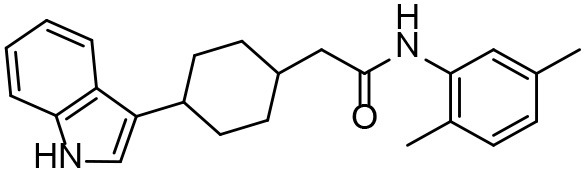	18.65 ± 0.87	22.72 ± 1.37	^*^
S16	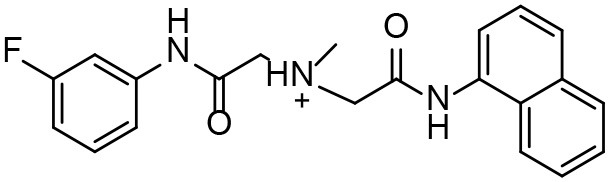	17.35 ± 0.20	21.00 ± 6.24	^*^
S19	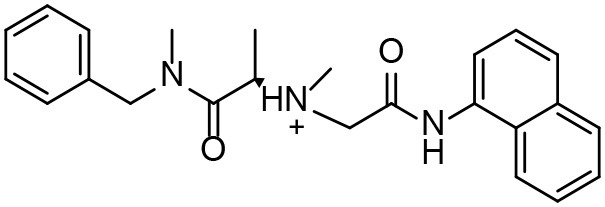	18.84 ± 1.70	22.54 ± 1.79	^*^
S28	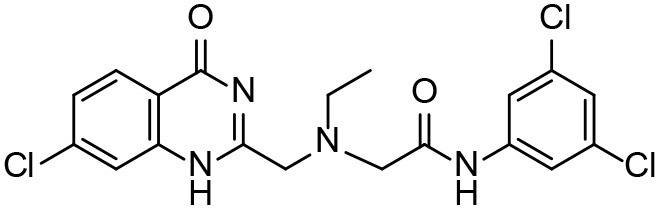	19.89 ± 0.31	20.99 ± 4.12	35.41 ± 0.62
S31	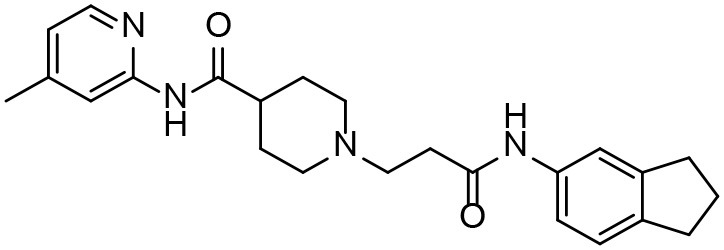	19.25 ± 0.80	ND	43.98 ± 1.38
S39	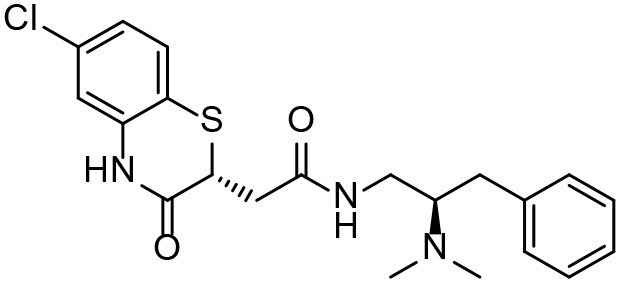	17.65 ± 0.74	ND	51.46 ± 3.34
S59	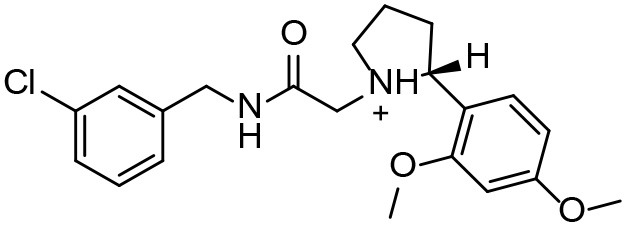	19.58 ± 0.56	ND	66.53 ± 2.99

* *The inhibition rates for compounds 6, 11, 16, and 19 were 24.65 ± 2.72%, 42.06 ± 1.78%, 34.56 ± 2.28%, and 36.51 ± 3.36% at 100 μM*.

Considering its effect on *E. multilocularis* PSCs and its cytotoxicity to cancer cells and non-carcinogenic mammalian cells, compound S6 was selected for additional investigation.

### *In vivo* Effects of the Candidate Compounds

**Figure 3:**
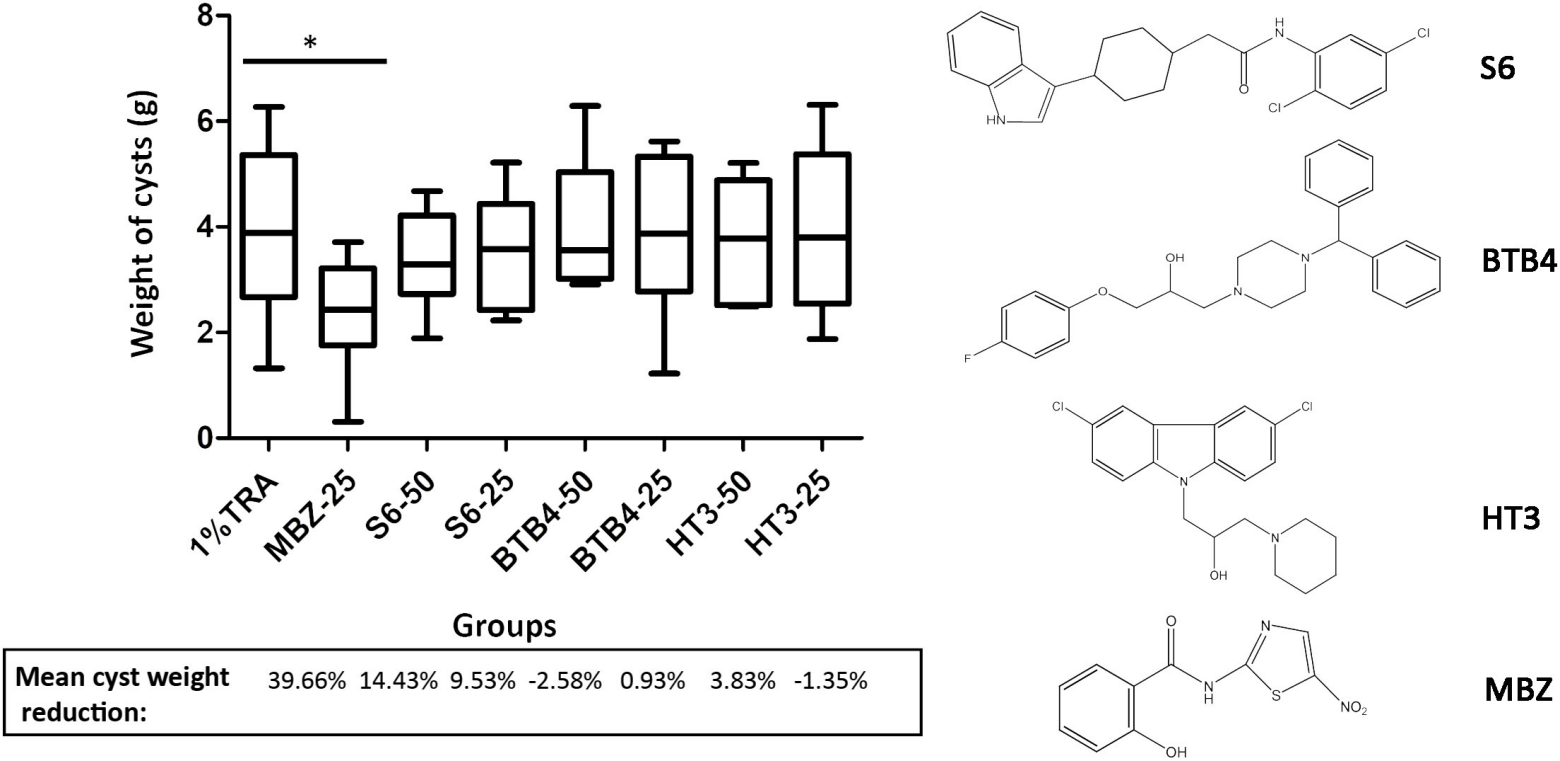
Treatment of mice secondarily infected with *E. multilocularis*. BALB/c mice were treated with S6, BTB4, and HT3 (25 mg/kg and 50 mg/kg), MBZ (25 mg/kg), or 1% TRA (control) for 28 days, *n* = 10. MBZ-25, BTB4-50, BTB4-25, HT3-50, and HT3-25 resulted in the deaths of 1, 4, 2, 3, and 2 mice, respectively. *p < 0.05.

We performed an *in vivo* pharmacodynamic evaluation of S6 and the previously reported active compounds, BTB4 and HT3 in mice infected with *E. multilocularis*. The inhibition rate of MBZ-25 was 39.66%, which was significantly lower than that of the control group (Figure 3). Although S6 at the two tested doses (50 and 25 mg/kg) inhibited the growth of *E. multilocularis* in mice (14.43 and 9.53%), there was no significant difference from the inhibition in the control group. Treatments with BTB4 and HT3 were confirmed to be ineffective in this study. In the 28-days treatment, MBZ-25, BTB4-50, BTB4-25, HT3-50, and HT3-25 resulted in the deaths of 1, 4, 2, 3, and 2 mice, respectively, while all the mice in the control and S6 groups remained alive during the experiment.

### Plasma Concentration and Pharmacokinetics of the Candidate Compounds in Mice

**Figure 4:**
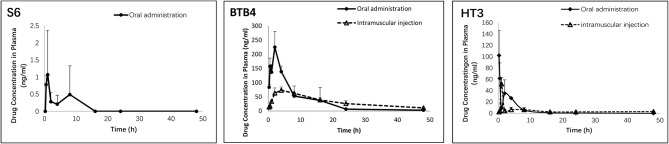
Concentrations of S6, HT3, and BTB4 in the plasma of mice after oral (50 mg/kg) and intramuscular (12.5 mg/kg) administration.

As depicted in Figure 4 and Table 4, the plasma concentrations of S6 in mice after administration by two methods were very low. The maximum concentration (C_max_) after oral administration was only 1 ng/ml, while the concentration after intramuscular administration was below the detection limit. After oral and intramuscular administration, the plasma concentration of BTB4 reached peaks of 225.13 ± 55.37 ng/ml and 86.39 ± 4.02 ng/ml (T_max_, maximum time) at 2 and 4.67 ± 3.06 h, respectively. After administration, the plasma concentration of HT3 rapidly reached its maximum. The T_max_ for oral administration was 0.33 ± 0.14 (C_max_ of 121.91 ± 42.63 ng/ml), and the T_max_ for intramuscular administration was 1.00 ± 0.00 (C_max_ of 39.59 ± 11.22 ng/ml). The area under the curve (AUC) and mean residence time (MRT), as other important pharmacokinetic parameters, for compound S6 were 5.10 ± 4.64 g/L^*^h and 4.34 ± 2.59 h, respectively. The AUC values for oral administration were higher than those for intramuscular administration for both BTB4 and HT3, but the intramuscular administration increased the residence time of the compound in mice.

**Table 4:** Pharmacokinetic parameters of S6, HT3 and BTB4 in the plasma of mice after oral (50 mg/kg) and intramuscular (12.5 mg/kg) administration.

**Parameters**	**S6**		**BTB4**		**HT3**	
	**Oral (50mg/kg)**	**Intramuscular (12.5 mg/kg)^a^**	**Oral (50mg/kg)**	**Intramuscular (12.5mg/kg)**	**Oral (50mg/kg)**	**Intramuscular (12.5mg/kg)**
AUC(0–t)/ g/L_*_h	5.10 ± 4.64	-	1692.29 ± 341.00	1589.90 ± 165.13	250.14 ± 32.49	191.91 ± 8.91
MRT(0–t)/h	4.34 ± 2.59	-	8.08 ± 1.55	16.02 ± 1.13	4.16 ± 0.85	18.12 ± 2.38
Tmax/ h	4.33 ± 3.51	-	2.00 ± 0.00	4.67 ± 3.06	0.33 ± 0.14	1.00 ± 0.00
Cmax/ng_*_mL^−1^	1.55 ± 1.01	-	225.13 ± 55.37	86.39 ± 4.02	121.91 ± 42.63	39.59 ± 11.22

a *The drug concentration was below the detection limit, so the pharmacokinetics were not calculated*.

## Discussion

This study was based on an analysis of the SAR of the pharmacophore model. The SAR describes the relationship between the chemical properties/three-dimensional structure of the compound and its biological activity, and researchers can identify the functional groups that play key roles in activities of small molecules (Ahamad et al., 2017). Any subsequent studies, such as the design of new compounds, structural modification of compounds, drug screenings and prediction of active compounds, are based on SARs. Quantitative structure-activity relationships (QSARs) and pharmacophore modeling are two important methods for SAR studies. QSARs are generally based on a series of compounds with the same framework and therefore can only guide the modification and activity prediction of compounds that are structurally similar (Verma et al., 2010; Yang et al., 2013). However, the pharmacophore model is derived from active compounds with different frameworks and generates the relevant pharmacophore characteristics to show the common features of active small molecules. That is, if a small molecule possesses the characteristics of a pharmacophore, then it may have a certain biological activity (Patel et al., 2002; Vlachakis et al., 2015; Gogoi et al., 2017). In our previous studies, we identified several structurally diverse active compounds out of in 130 amino alcohols, which is suitable for generating a pharmacophore model. Then we used the selected pharmacophore model to identify compounds with potential anti-echinococcal activity. Normally, the pharmacophore model can be generated by receptor-based and ligand-based methods. The former method relies on knowing the structure of the target (protein, DNA and so on) to analyse the interactions in the ligand-receptor complex (Gao et al., 2017; Kumar, 2018), while the latter method is based on the structures of the active compounds (Patel et al., 2002; Gogoi et al., 2017). Because the target of the series of amino alcohols involved in this study is unknown, a ligand-based approach was used to generate the pharmacophore model. In DS, model building includes two different pharmacophore modeling algorithms: generating common feature pharmacophore hypotheses from sets of known active ligands (HipHop) and also structure-activity relationship-based models when activity data is provided (HypoGen). The HypoGen module is for a group of compounds with different activities. With this method, the input training set compounds have activities in 4-5 orders s of magnitude, and each order has at least 3 compounds (Gupta et al., 2019). Unlike HypoGen, the HipHop algorithm does not consider the activity levels of the compounds in the training set. It only analyses only the three-dimensional spatial arrangement of the active compounds and uses an exhaustive search method to find pharmacophores that match the functional characteristics of the active compounds (Ataei et al., 2015; Fu et al., 2017; Che et al., 2018). Based on the results of *in vitro* drug screening of amino alcohols, we chose to establish a pharmacophore model using the HipHop module. Hence, a set of 6 training compounds was used to generate hypothetical pharmacophores using the HipHop algorithm. The structures were validated using the test set, and the best model (HipHop-Hypo03) was chosen to screen the ZINC database. Among the 176 compounds with fitness scores >4.0, 62 were tested on the parasites cultured *in vitro*. Ten of these compounds were identified as active, resulting in a screening hit rate of 16.13% (10/62). The virtual screening used in this study is an important example of the application of computer-aided drug design, including database searches based on pharmacophore models, QSAR models, structural similarity, molecular docking, pharmacokinetic properties and so on (Haga et al., 2016). These methods use computational methods and professional software to support hit finding and lead optimization in drug discovery. Generally, the hit rates are 5~20%, which is much higher than that of the high-throughput screening (Waszkowycz, 2008). Virtual screening has effectively shortened drug development time, reduced costs and improved efficiency, which can be very useful for the development of drugs to treat diseases that do not attract the attention of pharmaceutical companies. In recent years, computer-aided drug design has been applied to the discovery of anti-parasitic drugs (Postigo et al., 2010; Dube et al., 2012; Zhao et al., 2012; Pavadai et al., 2016; Vyas et al., 2016; Kagami et al., 2017; Araujo et al., 2018). Our study also indicated that virtual screening technology could play an important role in drug discovery for the treatment of echinococcosis.

Although the compounds in the training set used to generate pharmacophores were all amino alcohols, which are characterized by a hydroxyl group and an amino group connected by two carbon atoms, the structures of the 10 active compounds were all quite different. However, all of these compounds contained an amide bond, which is also present in BMZ and nitazoxanide, suggesting the importance of this moiety in affecting the activity against parasites. Further research on these active compounds showed that the LC_50_ values of PSCs were <20 μM, and 6 of them also affected cancer cells. Of these compounds, the low cytotoxicities of S5, S6, S11, and S16 against normal cells indicated good druggability. The structures of compounds S5, S6, and S11 were very similar, with the only difference being the position of the substituent on the benzene ring linked to -NH. As shown in Table 3, the substitution pattern of S5 resulted in a high toxicity, while S6 and S11, which contain two para-substituents, showed good activity and low toxicity. In addition, a –Cl substituent (S6) resulted in better efficacy than a –H substituent (S11).

Next, we selected compound S6 and two amino alcohols (HT3 and BTB4) to perform an *in vivo* pharmacodynamic study. Our preliminary results indicate that these two amino alcohols had better effects on cultured *E. granulosus* cysts *in vitro* and lower toxicity than the structures we reported (Liu et al., 2018). Hence, these two compounds, together with S6, were orally administered for 28 days at two doses of 50 and 25 mg/kg to mice infected with *E. multilocularis*. We found that although these compounds showed good effects against parasites *in vitro*, none were effective in mice secondarily infected with *E. multilocularis*. The death of experimental animals in the BTB4, HT3, and MBZ groups during the treatment period indicated that these compounds have notable toxicity.

In the development of new drugs, identifying an active compound is only the first step, and how to maintain the effective drug concentration and deliver the drugs to the target site is crucial and complicated. One difficulty in the development of anti-echinococcal drugs is that the drug must reach an effective concentration in the parasitic lesions through blood circulation, which is different from drugs for intestinal parasites that need to accumulate in only the intestine. Hence, high stability, high bioavailability, and the ability to penetrate the walls of cysts to achieve an effective concentration are requirements of good anti-echinococcal drugs. However, the existing active drugs/compounds, even the clinically used BMZ, do not have these characteristic and are limited by their poor absorption, so high-doses are still unknow, as is and long-term treatment is required in clinical use (Eckert et al., 2001). The physical and chemical properties that define good anti-echinococcal drugs are still unknown, as is how these drugs enter the cysts of parasites, whether by passive effusion or transporters. These uncertainties make screening criteria selection difficult. Hence, a pharmacokinetic study is necessary to observe how compounds behave *in vivo*. In this study, the concentration of S6 detected in plasma was very low. At many time points, the concentration was below the detection limit. For HT3 and BTB4, the drug concentrations did not reach the LC_50_. These results indicated that the poor performance *in vivo* may be related to the low drug concentration in plasma. In addition, the instability of S6 may also contribute to its poor drug efficacy because we found that if the drug and plasma samples were left overnight, the detectable concentration was greatly reduced. After analyzing the pharmaceutical parameters, we found that HT3 and BTB4 were not limited by absorption. Because they can rapidly enter the circulatory system and reach their peak concentration after oral administration, they are less affected by the first pass effect, suggesting that the drug's therapeutic effect can be imporved by increasing the dosage. However, their toxicities limit further studies on these two amino alcohols. For S6, the emphasis should be placed on increasing the administered dosage or structural modification after identifying the cause of its low plasma concentration, which will require additional experimental data.

## Conclusion

Based on the above discussion, the efficacy of pharmacophore-based screening was higher (positive screening rate of 16.13%) than that of traditional screening, which promising for discovery of novel drugs. Especially for echinococcosis, which is one of ten neglected tropical diseases, drug discovery research is hampered by the lack of both researchers and funding. We found that the failure of S6 in the treatment of AE at a dosage of 50 mg/kg per day for 28 days was closely related to its low concentration in plasma, which is the main reason for the treatment failure of many drugs *in vivo*. We carried out a virtual screening of compounds with drug-like properties to avoid this problem, but unfortunately, the active compound identified still showed poor druggability. However, we believe that once the screening database for screening is modified, the application of computer-aided drug design to the development of anti-echinococcal drugs will be very useful for finding novel anti-echinococcal compounds.

## Data Availability Statement

The datasets generated in this study are available by request from the corresponding author.

## Ethics Statement

The animal study was reviewed and approved by Animal care and all animal procedures were carried out in compliance with the Guidelines for the Care and Use of Laboratory Animals produced by the Shanghai Veterinary Research Institute. The study was approved by the Ethics Committee of the National Institute of Parasitic Diseases, Chinese Center for Disease Control and Prevention. The license number was IPD-2014-2.

## Author Contributions

HZ, YT, and CL developed the concept of the work. CL and ZX carried out the pharmacophore screening. CL, JYi, and JYa validated the effects of the compounds *in vivo* and *in vitro*. YT carried out the pharmacokinetics analysis. CL and JYi discussed and analyzed the results. CL wrote the paper.

### Conflict of Interest

The authors declare that the research was conducted in the absence of any commercial or financial relationships that could be construed as a potential conflict of interest.
