# Supplementary material for: Pharmacophore-Based Virtual Screening Toward the Discovery of Novel Anti-echinococcal Compounds
Source: Front Cell Infect Microbiol. 2020 Mar 20;10:118. doi: 10.3389/fcimb.2020.00118 (PMC7098963; doi:10.3389/fcimb.2020.00118)
Supplement: Supplementary file 1 [file Data_Sheet_1.doc]

Supplementary Material

# Supplementary Data

**Chemicals and materials**

Compound S6, HT3 and BTB4 were purchased from Enamine Ltd. (Kievska region, Ukraine). Praziquantel used as internal standard (IS) was purchased from Fluka (St. Louis, MO, USA). HPLC-grade formic acid was purchased from Dikma Technologies Inc. (Beijing, China). HPLC-grade methanol and acetonitrile were purchased from Emerck, Sinopharm Chemical Reagents Co., Ltd. (Shanghai, China). OASIS® HLB SPE 96-wells plates (30 mg) and OSTROTM 96-wells plates were purchased from Waters (Massachusetts, USA). All chemicals and solvents used were of analytical grade.

**HPLC Determination**

Chromatographic equipment and conditions

The HPLC–HRMS chromatographic system (ThermoFisher Scientific, Waltham, MA, USA) included an Accela 1250 pump, a PAL HTC autosampler, an Accela PDA detector and a Xcalibur data system. The assays of compounds were performed in a 3 μm Waters Alantics 2.1mm × 100 mm column (Massachusetts, USA) at 25 °C by elution with the mobile phase consisted of methanol (A) and water with 0.1% formic acid (B). An eluent flow rate of 0.35 ml/min was applied, and the gradient started at 35% B, increased linearly from 40% B (0.0–0.3 min) and further from 100% B (0.3–3.5 min), remained at 100% B (3.5-4.5 min), decreased linearly from 100-40% B (4.5–6.0 min), and finally remained at 40% B (6.0–6.5 min). The relative parameters were as follows: ESI ion source, 3.00 kV spray voltage, sheath gas (N_2_) and auxiliary gas (N_2_) 30 Arb and 2 Arb, respectively, capillary heater temperature 250 °C. The [M -H]+ quasi-molecular ion peaks of S6, BTB4, HT3 and praziquantel were detected at 402.11, 421.23, 377.12 and 313.19 mass-to-charge (m/z) ratio.

HPLC-HRMS Method Validation

*Specificity*

The HPLC method applied well for separation of tested compounds from IS with single peak (sFigure 1)


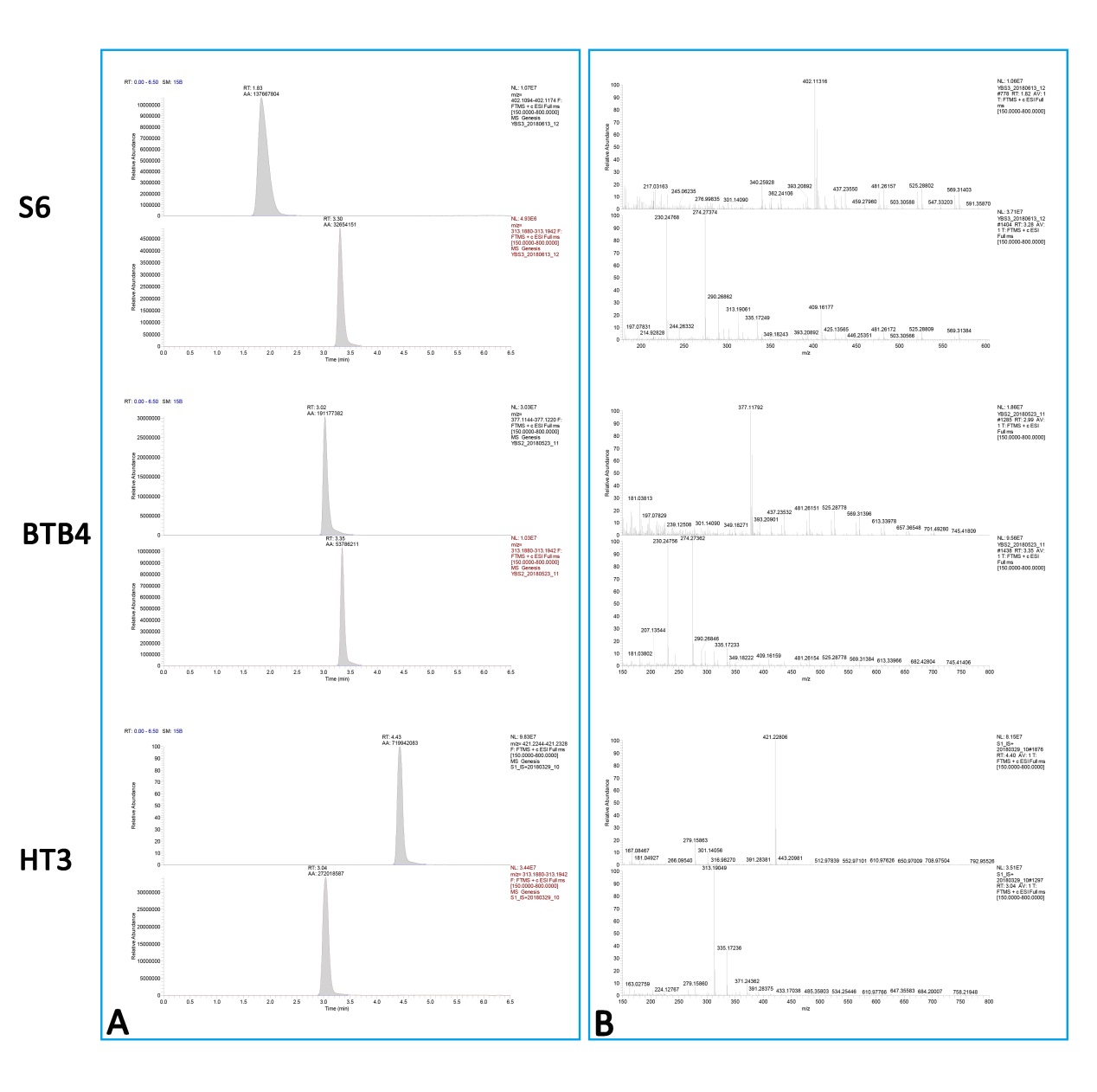


**SFigure 1 HPLC-HRMS performance of tested compounds and internal standard. A. RT = retention time at peak of the signal, AA = area under the curve; B. Full scan mass spectra, the ion peaks were the mass-to-charge ratios (m/z), S6, BTB4, HT3 and praziquantel were detected at 402.11, 377.12, 421.23 and 313.19 mass-to-charge (m/z) ratio.**

*Linearity*

The linearity of the calibration curve was determined by plotting the peak area ratio of tested compounds to IS (y) versus the nominal concentration (x) of tested compounds in plasma covering ranges from 0.01 to 10 μg/ml. The standard curves were calculated by using EXCEL 2010 and shown in sTable 1.

sTable 1. Standard curve of tested compounds in plasma

| Compounds in Plasma | Standard Curve | R^2^ |
| --- | --- | --- |
| S6 | Y=0.0004x+0.0008 | 0.9905 |
| HT3 | y=0.0309x | 0.9999 |
| BTB4 | y=0.0131x-0.032 | 0.9994 |

*Precision and accuracy*

To determine the within-day accuracy and precision of the method, tested compounds in plasma at three concentrations (0.04, 0.4 and 3.2 μg/ml) were individually analyzed five times in the same day. To determine the between-day accuracy, samples of plasma above were determined at five different days.

The intraday and interday precision at three concentrations were listed in sTables 2. Our results indicate that the accuracy of the current assay is suitable for further experiments.

sTable 2. Intraday and interday precision of the method for determination of tested compounds in plasma

| Tested compounds | Add concentration  (μg/ml) | Precision^a^ (%) | |
| --- | --- | --- | --- |
|  |  | Intraday (*n*=5) | Interday (*n*=5) |
| S6 | 0.04 | 2.39 | 2.03 |
|  | 0.4 | 1.99 | 1.21 |
|  | 3.2 | 1.46 | 0.68 |
| HT3 | 0.04 | 1.94 | 2.59 |
|  | 0.4 | 0.77 | 1.28 |
|  | 3.2 | 0.64 | 2.37 |
| BTB4 | 0.04 | 6.89 | 5.64 |
|  | 0.4 | 0.68 | 5.81 |
|  | 3.2 | 1.49 | 4.41 |

^a^Expressed as relative standard deviation (SD).

*Extraction recoveries*

The extraction recoveries of tested compounds were determined at 0.04, 0.4 and 3.2 μg/ml were added to mice plasma samples without adding IS. These samples were processed and dissolved by methanol with 10 μl IS. Then the absolute extraction recoveries were obtained by comparing the areas ratios of those samples to standard solution. The experiments were repeated three times and summaries in sTable 3.

sTable 3. Extraction recoveries of tested compounds in mice plasma (*n*=3)

| Tested compounds | Add concentration  (μg/ml) | Extraction recoveries (%) | |
| --- | --- | --- | --- |
|  |  | Mean | RSD |
| S6 | 0.04 | 62.32 | 5.27 |
|  | 0.4 | 79.71 | 4.23 |
|  | 3.2 | 76.49 | 5.87 |
| HT3 | 0.04 | 66.01 | 2.34 |
|  | 0.4 | 64.32 | 3.65 |
|  | 3.2 | 65.82 | 5.66 |
| BTB4 | 0.04 | 78.27 | 5.32 |
|  | 0.4 | 75.13 | 4.88 |
|  | 3.2 | 80.54 | 6.77 |

# Supplementary Figures and Tables

## Supplementary Figures

**
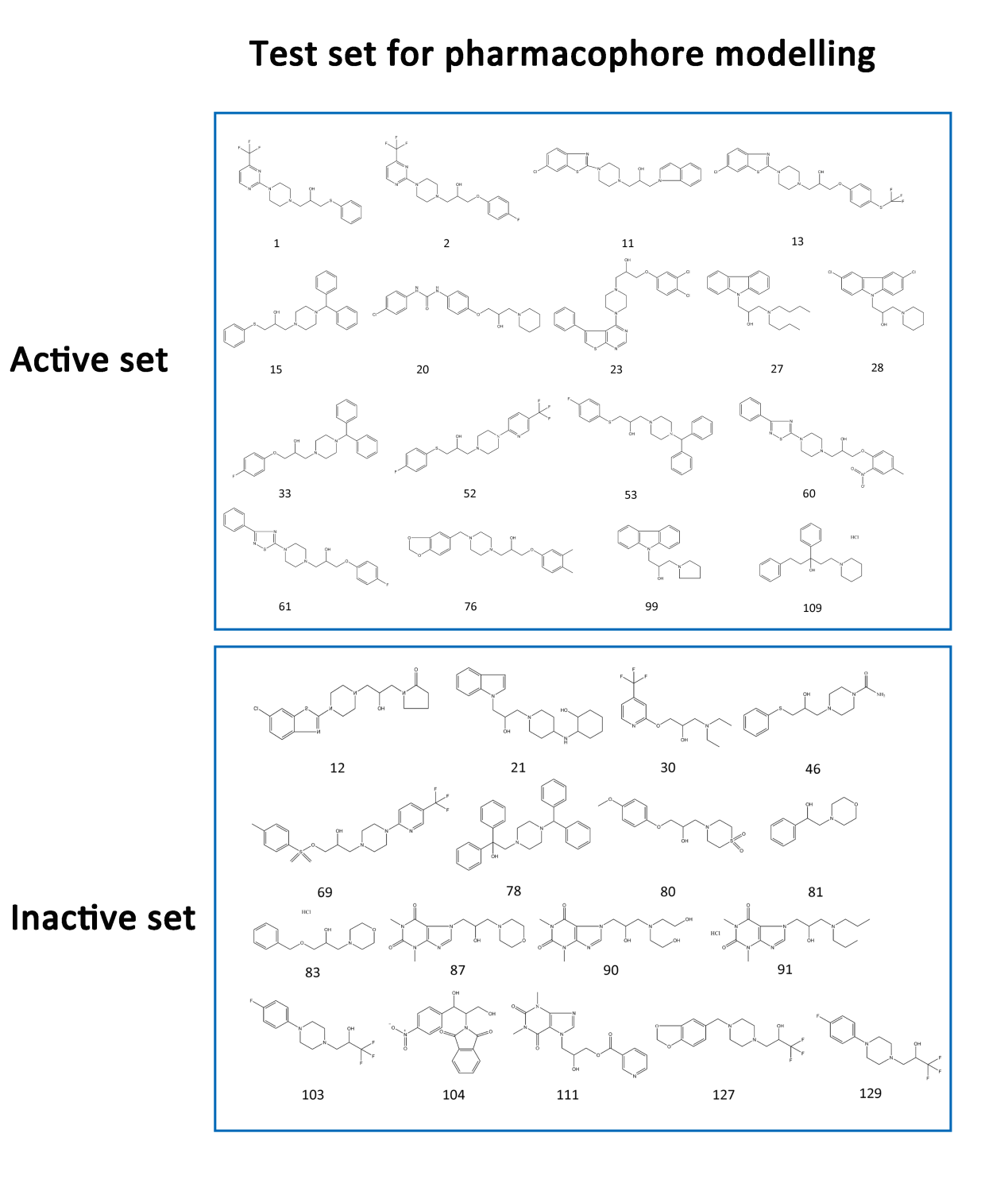
**

**Supplementary Figure 1.** The test set for pharmacophore modelling.

## Supplementary Tables

| **Supplementary Table 1**. The information of hits screened on *E. multilocularis* PSC *in vitro* | | |
| --- | --- | --- |
| **No.** | **ZINC ID** | **Fitvalues** |
| 1 | ZINC03333760 | 4.86 |
| 2 | ZINC22834510 | 4.74 |
| 3 | ZINC10139676 | 4.71 |
| 4 | ZINC12997266 | 4.69 |
| 5 | ZINC10139754 | 4.65 |
| 6 | ZINC10139689 | 4.65 |
| 7 | ZINC22843732 | 4.64 |
| 8 | ZINC35362524 | 4.62 |
| 9 | ZINC78950543 | 4.58 |
| 10 | ZINC07267733 | 4.56 |
| 11 | ZINC10139765 | 4.55 |
| 12 | ZINC52009249 | 4.54 |
| 13 | ZINC78950547 | 4.52 |
| 14 | ZINC69775772 | 4.51 |
| 15 | ZINC07267732 | 4.42 |
| 16 | ZINC03348408 | 4.41 |
| 17 | ZINC13071399 | 4.40 |
| 18 | ZINC12997400 | 4.40 |
| 19 | ZINC07267728 | 4.39 |
| 20 | ZINC11231705 | 4.37 |
| 21 | ZINC03377444 | 4.36 |
| 22 | ZINC35362526 | 4.36 |
| 23 | ZINC07454001 | 4.34 |
| 24 | ZINC12812480 | 4.33 |
| 25 | ZINC12997751 | 4.32 |
| 26 | ZINC15569177 | 4.31 |
| 27 | ZINC22932315 | 4.30 |
| 28 | ZINC13072152 | 4.30 |
| 29 | ZINC65475010 | 4.28 |
| 30 | ZINC52009245 | 4.27 |
| 31 | ZINC78748130 | 4.27 |
| 32 | ZINC58158667 | 4.26 |
| 33 | ZINC07267749 | 4.25 |
| 34 | ZINC13055992 | 4.25 |
| 35 | ZINC12334594 | 4.24 |
| 36 | ZINC13071397 | 4.23 |
| 37 | ZINC72290431 | 4.22 |
| 38 | ZINC03229012 | 4.21 |
| 39 | ZINC11603673 | 4.21 |
| 40 | ZINC08748371 | 4.19 |
| 41 | ZINC07267727 | 4.18 |
| 42 | ZINC13072150 | 4.18 |
| 43 | ZINC57352266 | 4.15 |
| 44 | ZINC72290377 | 4.15 |
| 45 | ZINC14075444 | 4.14 |
| 46 | ZINC12757641 | 4.13 |
| 47 | ZINC12886941 | 4.13 |
| 48 | ZINC28085089 | 4.12 |
| 49 | ZINC22881132 | 4.07 |
| 50 | ZINC13055994 | 4.07 |
| 51 | ZINC30363314 | 4.06 |
| 52 | ZINC76093195 | 4.06 |
| 53 | ZINC09460340 | 4.05 |
| 54 | ZINC00064559 | 4.04 |
| 55 | ZINC39726351 | 4.02 |
| 56 | ZINC32333123 | 4.02 |
| 57 | ZINC12992457 | 4.01 |
| 58 | ZINC22814243 | 4.01 |
| 59 | ZINC07741644 | 4.01 |
| 60 | ZINC12997605 | 4.01 |
| 61 | ZINC22056581 | 4.01 |
| 62 | ZINC97033359 | 4.00 |
